# Knowledge extraction for assisted curation of summaries of bacterial transcription factor properties

**DOI:** 10.1093/database/baaa109

**Published:** 2020-12-11

**Authors:** Carlos-Francisco Méndez-Cruz, Antonio Blanchet, Alan Godínez, Ignacio Arroyo-Fernández, Socorro Gama-Castro, Sara Berenice Martínez-Luna, Cristian González-Colín, Julio Collado-Vides

**Affiliations:** Centro de Ciencias Genómicas, Universidad Nacional Autónoma de México, Av. Universidad s/n, Colonia Chamilpa, Cuernavaca 62100, Morelos, Mexico; Centro de Ciencias Genómicas, Universidad Nacional Autónoma de México, Av. Universidad s/n, Colonia Chamilpa, Cuernavaca 62100, Morelos, Mexico; Centro de Ciencias Genómicas, Universidad Nacional Autónoma de México, Av. Universidad s/n, Colonia Chamilpa, Cuernavaca 62100, Morelos, Mexico; Centro de Ciencias Genómicas, Universidad Nacional Autónoma de México, Av. Universidad s/n, Colonia Chamilpa, Cuernavaca 62100, Morelos, Mexico; División de Posgrado, Universidad Tecnológica de la Mixteca, Carretera a Acatlima Km. 2.5, Huajuapan de León, 69000, Oaxaca, Mexico; Centro de Ciencias Genómicas, Universidad Nacional Autónoma de México, Av. Universidad s/n, Colonia Chamilpa, Cuernavaca 62100, Morelos, Mexico; Centro de Ciencias Genómicas, Universidad Nacional Autónoma de México, Av. Universidad s/n, Colonia Chamilpa, Cuernavaca 62100, Morelos, Mexico; Centro de Ciencias Genómicas, Universidad Nacional Autónoma de México, Av. Universidad s/n, Colonia Chamilpa, Cuernavaca 62100, Morelos, Mexico; Centro de Ciencias Genómicas, Universidad Nacional Autónoma de México, Av. Universidad s/n, Colonia Chamilpa, Cuernavaca 62100, Morelos, Mexico; Department of Biomedical Engineering, Boston University, 44 Cummington Mall, Room 403, Boston, 02215 MA, USA

## Abstract

Transcription factors (TFs) play a main role in transcriptional regulation of bacteria, as they regulate transcription of the genetic information encoded in DNA. Thus, the curation of the properties of these regulatory proteins is essential for a better understanding of transcriptional regulation. However, traditional manual curation of article collections to compile descriptions of TF properties takes significant time and effort due to the overwhelming amount of biomedical literature, which increases every day. The development of automatic approaches for knowledge extraction to assist curation is therefore critical. Here, we show an effective approach for knowledge extraction to assist curation of summaries describing bacterial TF properties based on an automatic text summarization strategy. We were able to recover automatically a median 77% of the knowledge contained in manual summaries describing properties of 177 TFs of *Escherichia coli* K-12 by processing 5961 scientific articles. For 71% of the TFs, our approach extracted new knowledge that can be used to expand manual descriptions. Furthermore, as we trained our predictive model with manual summaries of *E. coli*, we also generated summaries for 185 TFs of *Salmonella enterica* serovar Typhimurium from 3498 articles. According to the manual curation of 10 of these *Salmonella typhimurium* summaries, 96% of their sentences contained relevant knowledge. Our results demonstrate the feasibility to assist manual curation to expand manual summaries with new knowledge automatically extracted and to create new summaries of bacteria for which these curation efforts do not exist.

**Database URL:** The automatic summaries of the TFs of *E. coli* and *Salmonella* and the automatic summarizer are available in GitHub (https://github.com/laigen-unam/tf-properties-summarizer.git).

## Introduction

To gain a better understanding of bacterial transcriptional regulation, complete descriptions of properties of transcription factors (TFs) are highly valuable. TFs are proteins that bind DNA and regulate promoter activity; they affect transcription initiation by repressing or activating gene expression. Several properties of these proteins are used to understand how they work and their effect on cell growth and cell division.

TF properties related to regulation are described mainly in biological databases containing curated knowledge from the biomedical literature. For example, knowledge of TFs of *Escherichia coli* K-12 not only in structured records but also in textual format as summaries manually curated generated by curators at UNAM are available both in RegulonDB (1, 2) and in EcoCyc (3). Textual descriptions of TFs have been published also for eukaryotes (4).

However, the manual creation of those textual descriptions (manual summaries) involves demanding and time-consuming efforts, because properties of TFs are published, gradually, in many scientific articles. The time and effort to obtain knowledge of TF properties increase when new article collections are taken into consideration to expand a database or when a new database for a different organism is created. No doubt, biocuration projects can benefit using automated approaches to assist curation by extracting specific knowledge from large article collections.

To face this challenge in the area of biomedical natural language processing (BioNLP), different approaches to detect and extract knowledge from scientific article collections have been developed. Tasks related to information extraction, such as biological named entity recognition (NER) and relation extraction, have been prioritized by the BioNLP community (5). In addition, approaches for automatic text summarization (ATS) of biomedical document collections have been developed (6, 7). ATS is a natural language processing (NLP) technique for developing systems (automatic summarizers) that deliver a compressed version (automatic summary) of a document collection by selecting the most relevant content (8).

Here, we show an effective strategy of knowledge extraction by ATS to assist curation of manual summaries of TF properties of bacteria. We developed an approach to obtain user-oriented multi-document extractive summaries based on automatic classification, a supervised machine learning approach, which has been used to determine relevant content in biomedicine (9–12).

In a previous report (13), we demonstrated the feasibility of this approach by obtaining automatic summaries with high recall for only two TF properties: structural domains and biological processes of the regulated genes. In the present study, we address some limitations of the previous study.

First, we addressed the problem of imbalanced classes and made changes in our previous supervised learning and ATS strategies. Second, while in the previous study we only tested our approach with five TFs of *E. coli*, here we show the performance of our approach by evaluating our method on 177 manually curated summaries of TFs of *E. coli.* Subsequently, we processed a collection of 3498 articles related to transcriptional regulation of *Salmonella typhimurium* and generated summaries for 185 TFs. This may be seen as a knowledge transfer approach with the possibility of being used for diverse bacteria.

Two curators evaluated 10 summaries for *S. typhimu-rium* by categorizing the relevance of their sentences (relevant/not relevant) obtained from a subset of 550 articles. Of the sentences in these summaries, 96% were relevant for describing the TF, and 65% were related to the correct property. The automatic summaries of the TFs of *E. coli* and *Salmonella* are available through GitHub (https://github.com/laigen-unam/tf-properties-summarizer.git). Based on these results, we corroborated that our approach for knowledge extraction of TF properties can assist curation of new summaries in other bacterial species.

## Materials and methods

### Manual summaries as training data

**Figure 1.**
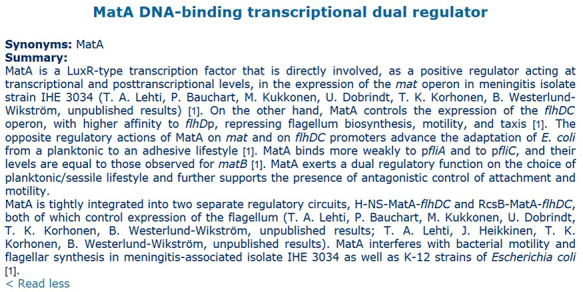
Manual summary of MatA from RegulonDB. Numbers in brackets are citations for the articles employed to elaborate the summary.

The team of curation at the Center for Genomic Sciences has >20 years of experience devoted to curation of transcriptional regulation and operon organization of *E. coli* K-12, extracting knowledge from original papers and making the product of this work available both in RegulonDB (1, 2) (http://regulondb.ccg.unam.mx/) and in EcoCyc (https://ecocyc.org/). One of the most time-consuming efforts by curators is the generation of TF summaries, which gather in text format a summarized description of evolutionary, functional, mechanistic and structural properties of TFs and of the regulated genes and the role of their regulation in the physiology of the cell. These summaries require curators to read a good number of papers to generate them (13). An example of these textual descriptions, the manual summary from RegulonDB of the TF MatA, is shown in Figure 1.

The TF properties included in manual summaries of RegulonDB that we considered in our study were the following:

(i) Transcriptional activity (ACT): the active and inactive conformations of the TF, if the TF is a repressor, activator or dual transcriptional regulator; the growth conditions under which the TF is activated or inactivated and if the TF has an effector molecule.
(ii) Structural domain (DOM): the number, name and size of the structural domains constituting the TF.
(iii) Evolutionary properties (EVO): information about evolutionary properties of the TF, such as its membership within an evolutionary family of TFs.
(iv) Regulated processes (RP): the biological processes in which the regulated genes are involved.
(v) TF binding sites (SITE): information about the TF binding sites, such as size and the symmetry of the consensus sequence.
(vi) Transcription unit (TU): information about the organization of the TU (operon) that contains the gene encoding the TF.

We employed these manual summaries as training data for an automatic summarizer that extracts relevant sentences describing these TF properties from article collections. To compile such data, we manually classified the sentences of 177 summaries of TFs of RegulonDB for one or more of the six TF properties. To classify the sentences, we tagged specific relevant information associated with each TF property using XML tags (see a description of this information in Supplementary Table S1 and an example of a tagged manual summary in Supplementary Figure S1). By tagging this information, we defined several resources (e.g. dictionaries of biological entities and lists of keywords) that were used to enrich the input features into the supervised learning algorithm (see section “Supervised learning approach”). For the properties DOM and RP, we took advantage of the sentences manually classified in our previous study.

We obtained a training data set with 2244 sentences in total formed by 6 subsets of sentences from all summaries, 1 per TF property. Positive examples were those pertaining to the TF property, while negative examples were all the remaining sentences. The data sets showed imbalanced classes, with a much smaller number of positive versus negative cases, especially for SITE, TU and EVO (Table 1). To deal with this problem, we tested some automatic strategies in our supervised learning approach.

**Table 1.** Description of the training data set of manually classified sentences, with the percentage of the positive class for each TF property

Property	Positive examples	Negative examples
ACT	273 (12%)	1971
DOM	335 (15%)	1909
EVO	164 (7%)	2080
RP	261 (12%)	1983
SITE	127 (6%)	2117
TU	150 (7%)	2094

### Approach for knowledge extraction of TF properties

We approached the problem of knowledge extraction of TF properties from the literature as a problem of automatic user-oriented multi-document extractive summarization. In extractive summarization, an automatic system (summarizer) selects the most relevant sentences from an input document collection as a summary. This strategy is different from abstractive summarization, in which the summarizer collects the relevant ideas from the input documents and delivers a summary formed by sentences generated automatically using tasks such as text generation or sentence compression. Moreover, our summarization approach was user-oriented, because specific information was required to be summarized, that is, the TF properties.

To extract the relevant sentences associated with each TF property, we used automatic classification, a supervised learning approach in which a machine learning algorithm compiles from human validated examples a predictive model to assign a category (class label) to new unclassified examples. In our previous study (13), we demonstrated that a predictive model trained with manual summaries can recover relevant sentences from an article collection, carrying the same knowledge contained in manual summaries.

#### Supervised learning approach

**Figure 2.**
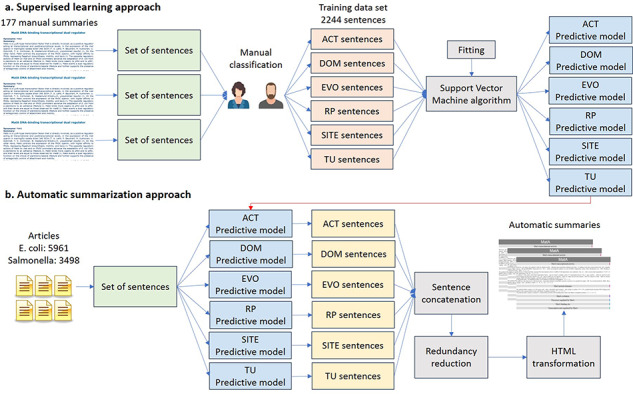
Automatic approach for knowledge extraction of TF properties. (a) Supervised learning approach. (b) Automatic summarization.

We trained six binary classifiers with a support vector machine (SVM) algorithm, one for each type of property, using combinations of different features of the 2244 sentences of the training data sets (Figure 2a). These binary classifiers determined when a sentence belongs to a TF property (positive class) or not (negative class). As we used different classifiers for each TF property, a sentence could be classified with more than one TF property.

In traditional machine learning approaches, features are determined by the human analyst and then extracted from the examples of the training data. Here, we employed sentences of manual summaries as training examples and explored combinations of some features of these sentences to determine the best predictive model. Instead of using words as features of sentences, we used lemmas, that is, the normalized versions of words. For example, instead of the words ‘activates’, ‘activating’ and ‘activated’, we used the lemma ‘activate’, that is, the infinitive form of the verb. In addition, we used the part-of-speech (POS) tags of the words of the sentences, that is, tags encoding a grammatical category, such as VBZ for a verb third person singular present, or NN for a noun. To obtain lemmas automatically, we employed BioLemmatizer (14), and for POS tags, we employed the Stanford CoreNLP (15).

We enriched linguistic features (lemmas and POS) with tags of biological information using a dictionary-based NER approach. In this approach, words are looked for in dictionaries of biological terms and entities; if the word appears, its tag is returned. For example, the word ‘regulated’ can be tagged as ‘verb of regulation’ with the tag ACTREG, and HipAB can be tagged as a ‘transcription factor’ with the tag ACTTF. This biological information was tagged in sentences of manual summaries and article collections, and then, we used dictionaries of biological terms and entities for *E. coli* and for *Salmonella*. In the case of the biological terms, most of them were utilized for both bacteria.

For the ACT property, we tagged names of TFs, effectors, growth conditions, conformations, and verbs of regulation, as well as terms related to effectors, conformations and regulation. For DOM, dictionaries of TFs and protein domain characteristics were used, including structural motifs, domain families and their functions, as well as a set of related terms frequently mentioned in manual summaries. For EVO, we employed dictionaries of evolutionary families and domain positions, as well as a set of terms used to describe structure conservation. For RP, we used a dictionary of biological processes and a set of frequent terms related to regulated processes. For SITE, we tagged two sets of terms associated with this property: terms related to binding site symmetry and terms related to DNA sequence length description. Also, to tag the spatial arrangement of binding sites, a regular expression was used to identify DNA motifs. Finally, for TU, we used dictionaries of genes and TUs, plus terms describing the organization, regulation and localization of the TUs. More detail about the different sources of information for the two bacteria is included in Table S2 in Supplementary material.

To feed TF property classifiers with training data, we explored three combinations of features for sentence representation: (i) lemmas, POS and NER (lemma POS NER), (ii) lemmas and NER (lemma NER) and (iii) lemmas and NER, but substituting lemmas when they had NER tags (NER for lemma). Examples of these combinations are shown in Table 2. In addition to individual features, we tested groups of two or three joint features to create features called n-grams. All combinations were vectorized in three different ways: presence or absence of the feature (binary), term frequency - inverse document frequency (TF-IDF) weight and binary TF-IDF weight (after applying TF-IDF, all values above 0 were turned to 1). The combination of features NER for lemma and the vectorization with binary TF-IDF weight were not tested in our previous study.

**Table 2.** Sentence representations employed in supervised learning using combination of features

Combination	
of features	Sentence representation
Original sentence	HipAB toxin-antitoxin system appears to be regulated at level of HipB stability.
lemma POS NER	HipAB toxin-antitoxin system appear to be regulate at level of HipB stability. ACTTF NN NN VBZ TO VB ACTREG IN NN IN ACTTF NN
lemma NER	HipAB toxin-antitoxin system appear to be regulate at level of HipB stability. ACTTF ACTREG ACTTF
NER for lemma	ACTTF toxin-antitoxin system appear to be ACTREG at level of ACTTF stability.

NN = noun;  VBZ = verb   third   person   singular present; TO = infinitival to; VB = verb base form; IN = conjunction, subordinating, or preposition; ACTREG = verb of regulati- on;ACTTF = name of TF.

In this study, we also included some strategies to face the three machine learning challenges observed in our data sets: (i) imbalanced classes, (ii) high dimensionality and (iii) high sparsity. The problem of imbalanced classes occurs when the number of examples of the positive class is much smaller than the number of examples of the negative class (16), which is our case (section “Manual summaries as training data”). High dimensionality and sparsity are common in textual data (17), due to the fact that the number of different features employed to vectorize examples is very large, and any given subset of them appears in only a few examples.

To deal with the imbalanced classes, we tested four under-sampling techniques to balance the examples of the two classes by eliminating examples of the majority class: random under-sampling (RandomUS), Tomek’s link, one-sided selection and instance hardness threshold. We employed the Python imbalanced-learn application programming interface, which includes implementations of these strategies (18). In the RandomUS technique, a subset of examples of the majority class is randomly eliminated so that the same number of examples as the positive class is included in both classes. Tomek’s link technique is based on searching for all pairs of nearest neighbor vectors, which represent sentences, based on a distance metric where each vector belongs to a different class; this pair is called a Tomek’s link (19). Then, all examples of the majority class of the Tomek’s links are removed.

For the one-sided selection technique, the objective is to select a representative subset of the examples of the majority class (16). Borderline and noisy examples are eliminated by removing examples of the majority class from Tomek’s links, and redundant examples are eliminated by selecting a subset of examples of the majority class that can classify correctly the original data sets with a one-nearest-neighbor rule. The aim of the instance hardness threshold technique is to remove a subset of examples from the majority class that are hard to classify by a given learning algorithm used as a user-defined parameter (20); for this, we selected the SVM algorithm. These examples have the lowest probabilities to belong to the positive class, which is used as a criterion to remove them from the training data. With this method, we cannot establish the number of final examples of the majority class.

It has been emphasized in machine learning research that general performance scores, especially accuracy, are not suitable to evaluate classifiers when the imbalanced classes problem is present (21). The geometric mean (G-mean) has been proposed as a proper evaluation score for the imbalanced class problem (16, 22). Hence, we decided to employ such a score (Equation (1)) as the general evaluation score for cross-validation of all our experiments. This measure expresses the balance between accuracy on positive examples (sensitivity; Equation (2)) and accuracy on negative examples (specificity; Equation (3)), based on the four combinations of correctly and incorrectly classified examples: true positives (TPs), true negatives (TNs), false positives (FPs) and false negatives (FNs). Therefore, the G-mean takes into account the performance of the two classes, so that a low performance score for the positive class gives a low G-mean value even though the negative class was correctly classified.

(1) \begin{equation*}G - mean = \sqrt {Sensitivity \times Specificity} \end{equation*}

(2) \begin{equation*}Sentitivity = {{TP} \over {TP + FN}}\end{equation*}

(3) \begin{equation*}Specificity = {{TN} \over {TN + FP}}\end{equation*}

In order to increase the ability of the trained classifiers to detect varied forms of sentences in the future (i.e. generalization), we employed a 10-iteration (10-fold) stratified cross-validation strategy, instead of splitting the data set into two parts (training and validation). In this way, we trained with nine iterations and validated with one. At the end of 10 iterations, we used all positive instances for training and validation. In addition, we adapted this strategy to be performed in accordance with the class imbalance by applying under-sampling of the negative class exclusively over the nine training iterations and excluding the validation iteration (23).

To deal with the high dimensionality and sparsity of vectors representing sentences, we tested two approaches: the singular value decomposition (SVD) approach and the feature selection via the *χ*^2^ (chi-squared) statistic approach. The SVD is one of the most frequently employed approaches for dimensionality reduction in NLP (17, 24). This approach performs a matrix decomposition to obtain a low-rank approximation using only the number of highest singular values, which corresponds to the final dimensions. We explored dimensionality reduction to 200 and 300 dimensions. Feature selection is an approach to select a subset of features to reduce the size of training data by eliminating noisy features. To use the *χ*^2^ approach for feature selection (17), features and classes are considered events. Then, if a feature and a class are significantly dependent, the approach selects the feature as part of the subset of helpful features. We tested this approach by selecting 1000, 800 and 500 features.

We used the SVM algorithm to obtain the six binary classifiers (25, 26). In a vector space where training data are represented, the algorithm finds the best decision function that separates vectors representing sentences of the two classes. To do that, the algorithm determines a subset of sentences for each class, which are the hardest to distinguish. Their vector representations are known as support vectors and are used to build the decision function, which is unique for a training set. This ensures that the remaining sentences are easier to classify. This algorithm has been used successfully in many NLP tasks (17).

Regarding the user-defined parameters of the SVMs, we tested three kernels to provide additional adaptability to the decision function: linear, radial basis function (denoted as rbf) and polynomial (denoted as poly). We also tested other user-defined parameters like C (which adjusts the variability of the training), and degree and gamma (both of which affect the behavior of the different kernels) by using a grid search. We also examined training the SVMs with class weighting as an additional strategy to address the imbalanced data problem. Using this strategy, classification mistakes of the minority class are penalized by modifying the C hyperparameter for this class. We utilized the scikit-learn Python machine learning library (http://scikit-learn.org/stable/), which includes specific objects and methods to perform a grid search cross-validation training strategy of a support vector classifier (SVC). In Supplementary Table S3, we show a general description of the experimental setup, including the tested values of the different aspects employed for training the six classifiers.

**Figure 3.**
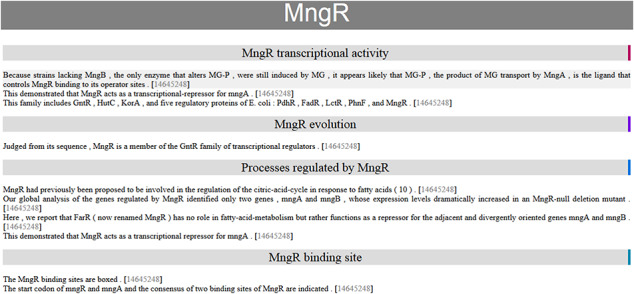
Automatic summary of MngR in HTML format, showing sentences classified into TF properties and with the PubMed ID hyperlink to the article in the PubMed database. The automatic summary is organized by separating properties in different sections, and sentences are sorted by similarity, so that the curator finds related information together.

#### Automatic summarization approach

To obtain the automatic summaries from the 5961 articles on *E. coli* (including those articles used by curators to write the manual summaries) and the 3498 articles on *Salmonella*, the six classifiers were applied to the set of sentences of these articles (Figure 2b). The classified sentences were then concatenated according to the classified TF property to form the automatic summary. To try to guarantee that the sentences in the final summary were related to the TF, only those sentences containing the name of the TF were saved. For *E. coli*, we generated an additional version of automatic summaries using only those articles employed by curators to write the manual summaries, in order to assess the performance of our summarizer in article collections related to a specific TF (TF-related articles).

In multi-document text summarization, the automatic summary commonly contains duplicated and highly similar sentences, especially when the document collection refers to a specific topic. Hence, we implemented an approach to reduce redundant information in the final summary by using a hierarchical clustering based on cosine similarity of sentences, a standard similarity measure in NLP tasks (17) with values from 0 up to 1. In detail, from a binary TF-IDF vector representation of the set of sentences of each property in the automatic summary (without stop words), we calculated a cosine similarity matrix, which was used to cluster sentences with the average linkage method (27).

Hierarchical clustering put similar sentences together, so we kept the first sentence and discarded those sentences highly similar (cosine similarity up to 0.8) until we find a dissimilar sentence (cosine similarity < 0.8). Then, we kept this sentence and repeated the same procedure until processing all sentences. At the end, automatic summaries have only dissimilar sentences selected according to the order given by the hierarchical clustering. This strategy was applied to automatic summaries for *E. coli* and *Salmonella* before evaluation.

One of the open challenges for BioNLP is to have impact in real curation scenarios by making extracted knowledge accessible and usable for curators. Since our method does not achieve the same final performance as manual curation, we decided to implement an assisted curation strategy. Thus, our final automatic summaries are created in HTML format, facilitating use by curators to create the final publishable summaries. Moreover, the HTML-formatted automatic summaries are organized by separating properties into different sections and sentences are sorted by similarity, so the curator finds related information together. In these HTML summaries, instead of discarding redundant (highly similar) sentences, we decided to keep them in hidden format with the possibility for the curator to see them. In addition, each sentence includes the PubMed ID for the article from which it was extracted, which is a hyperlink to the article in the PubMed database.

The HTML automatic summary of MngR is shown in Figure 3 as an example of the output of our approach. This contains information related to four properties. There is a sentence in two sections (‘transcriptional activity’ and ‘processes regulated’) because our approach assigns one or more classes to each sentence.

### Evaluation of knowledge extraction

To appraise the amount of knowledge that our approach retrieves in an automatic summary from an article collection, we employed Recall-Oriented Understudy for Gisting Evaluation (ROUGE) (28). ROUGE is a widely used evaluation method for automatic summarization based on the overlapped word statistics between a manual and an automatic summary (6, 7). ROUGE scores range from 0 to 1, where 1 means capturing all relevant information, in our case, knowledge of bacterial TF properties. A ROUGE score can be seen as a percentage of the knowledge shared between a manual and an automatic summary.

The ROUGE score can be calculated as either a recall or a precision score. On the one hand, the score for ‘ROUGE recall’ measures how much relevant information of the manual summary is contained in the automatic summary. To calculate this score, the number of overlapped words between the manual and the automatic summaries is divided by the total number of words in the manual summary. On the other hand, the score for ‘ROUGE precision’ measures how much relevant information is in the automatic summary with respect to the expected information of the manual summary. For precision, the number of overlapped words is divided by the total number of words in the automatic summary.

Because our aim was to assist creation of summaries by curators (assisted curation), we preferred to extract most of the knowledge from the article collection (high recall), instead of delivering short automatic summaries (high precision). Compact automatic summaries containing abundant knowledge are ideal, but this requires more elaborate approaches for compressing the final automatic summary. Consequently, we based our evaluation on ROUGE recall scores. For a better estimation of the extracted knowledge, we discarded very common words (stop words) from the evaluation. From the variety of ROUGE methods, we used those evaluating overlapping of individual words (ROUGE-1), which we previously showed is a reliable approach to evaluate user-oriented ATS (13).

#### Evaluation for E. coli

As the manual summaries included the PMIDs that were used for writing them, we were able to generate an automatic summary for each TF using only the corresponding PMIDs (TF-related articles). In addition, we generated summaries using the complete collection of 5961 articles that has been curated to date for RegulonDB. By comparing these two kinds of automatic summaries, we explored if using the complete article collection allowed us to extract more knowledge of the properties of a specific TF than using only those TF-related articles. We evaluated all summaries using ROUGE-1 recall.

#### Evaluation for S. typhimurium

Since we also wanted to study the extraction of knowledge of TF properties of one bacterium employing the predictive model trained with another one, we generated the automatic summaries of 185 TFs of *S. typhimurium* from a collection of 3498 articles, applying the predictive model utilized to generate the automatic summaries of *E. coli*. The collection of articles related to *Salmonella* was provided also by the team of RegulonDB.

As we did not have manual summaries of TF properties for *Salmonella*, we were not able to achieve an automatic evaluation with ROUGE. Therefore, in order to provide insight into how useful the obtained summaries were, we asked two curators of the team of RegulonDB to evaluate the sentences of 10 summaries in terms of relevance. From a subset of summaries generated automatically, we selected the shortest summaries that included at least one sentence per property. The two curators evaluated the same summaries and we reported the average score. The curators decided if a given sentence was relevant for describing knowledge of the corresponding property (yes/no) and if the sentence was relevant for describing general knowledge of the TF (yes/no), regardless of the corresponding property. The first type of relevance takes into consideration not only if the sentence is relevant but also if our approach assigns the property correctly. The evaluated summaries are available through GitHub (https://github.com/laigen-unam/tf-properties-summarizer.git).

## Results

### Knowledge extraction for *E. coli*

#### The approach recovered most of the knowledge of the manual summaries

**Figure 4.**
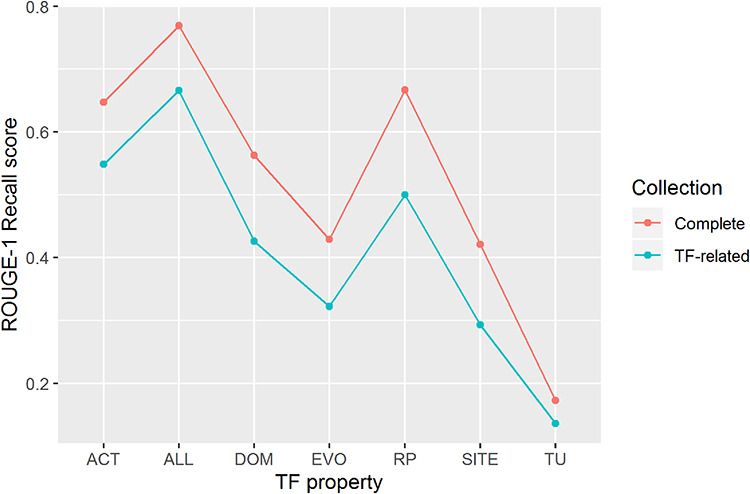
ROUGE-1 recalls scores obtained by automatic summaries without stop words for all properties (ALL) and for each property. Automatic summaries were created using the complete article collection (Complete) and using only TF-related articles (TF-related).

We were able to recover 77% of the knowledge of the TF properties of 177 TFs (ROUGE-1 recall median score of 0.77) by processing the complete article collection of RegulonDB (Figure 4). We highlight that the score was obtained without considering stop words (highly frequent words, such as prepositions, articles and common verbs), so this evaluation is based mostly in biomedical terms and verbs that overlap between the automatic and the manual summaries.

Processing only TF-related articles (those articles used by curators to produce the manual summaries), again without considering stop words, our approach extracted 67% of the knowledge of all TF properties of 170 TFs (ROUGE-1 recall median score of 0.67). In this case, 7 of the 177 TFs were excluded from the evaluation because their related articles were not available.

For individual TF properties, we observed the best performance for knowledge extraction of biological processes of genes regulated by the TF (RP), a property that describes delimited information, and transcriptional activity of the TF (ACT), a property that includes assorted information (Figure 4). On the contrary, knowledge extraction of the TU that contains the gene encoding the TF got the lowest median score.

#### The approach extracted knowledge from articles not used in manual summaries

Manual summaries were written based on a median of 8 articles, while the automatic summaries were generated from a median of 15 articles. Our approach extracted knowledge from a median of 12 additional articles that were not utilized to write the manual summary. This confirmed the helpfulness of our approach to extract additional knowledge to assist curation. A median of one article from those employed in the manual summary was missed in the automatic summary.

#### The approach extracted additional properties that were not present in manual summaries

A remarkable result was that by using our approach we were able to extract additional properties that were not present in the manual summary for 71% of the 177 TFs of *E. coli* (Table 3). These additional properties may be considered by curators to expand TF summaries currently present in RegulonDB. A median of five properties was detected in both the automatic and manual summaries. It is important to note that our approach did not extract 4 properties for 1 TF, 2 properties for 5 TFs and 1 property for 19 TFs; only 14% of the TFs did not have missing properties.

**Table 3.** Percentage of TFs with additional properties recovered by our approach

No. of additional properties recovered	TFs	% of total TFs
5	2	1%
4	9	5%
3	16	9%
2	36	20%
1	63	36%
0	51	29%
	177	100%

#### Automatic summaries were larger than manual ones

**Figure 5.**
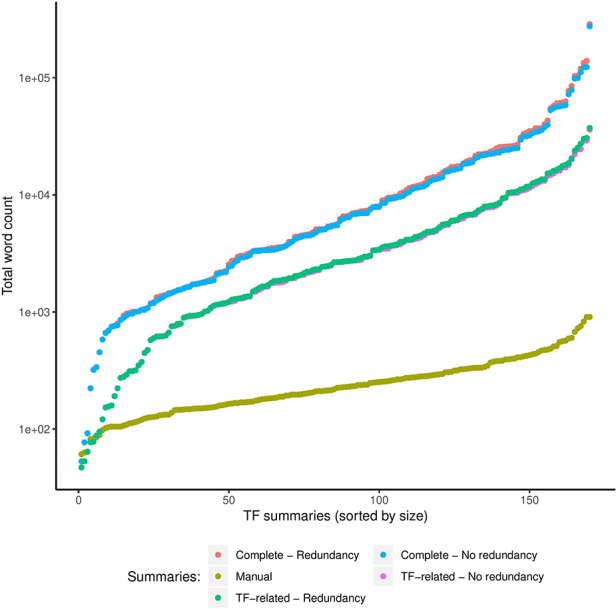
Comparison of sizes in total words (log scale) of manual and automatic summaries. We show the size of automatic summaries generated from the complete article collection and from the TF-related articles. Also, we show the size of the automatic summaries with (redundancy) and without redundant sentences (no redundancy).

As expected, our approach generated larger automatic summaries using the complete article collection (Complete) compared with the size in total words of the manual summaries (Figure 5). Even automatic summaries created with only TF-related articles were larger than manual summaries. However, with respect to the complete article collection that we summarized, the median compression rate of the automatic summaries was 0.01%, that is, automatic summaries were on average 0.01% the size in words of the complete collection. For the TF-related articles, the median compression rate was 8%, with a standard deviation of 6%.

#### Reductions in sizes of automatic summaries by redundancy reduction were minimal

Reductions in sizes of automatic summaries by our clustering-based approach for redundancy reduction were minimal. As shown in Figure 5, the reduction of the word count computed on automatic summaries that included redundant sentences (redundancy) versus summaries without redundant sentences (no redundancy) was far from significant. Removing redundant sentences helps to synthesize the information presented to the curator while keeping the most relevant information. In the future, we will explore more elaborate approaches to compress the final summary beyond the dropping of very similar sentences (redundant information).

### Knowledge extraction for *Salmonella* resulted in a high number of relevant sentences

Manual evaluation by two curators of 10 automatic summaries generated for *Salmonella* from a subset of 550 articles showed that 96% of the sentences were relevant sentences to describe properties of a TF. However, only 65% of the sentences were classified with the correct property (Table 4).

### The supervised learning approach learned to classify sentences for TF properties

The results for knowledge extraction of the TF properties depended on the performance of our supervised learning approach to classify sentences associated with each TF property in the manual summaries. We observed that our approach obtained a good performance according to the G-mean scores obtained in the cross-validation (Table 5). SVD transformation to reduce to 200 dimensions helped to train models to classify better for almost all models.

**Table 4.** Percentage of correctly classified relevant sentences of 10 automatic summaries on *Salmonella* evaluated manually by two curators

Property	Correctly classified
ACT	80%
DOM	41%
EVO	76%
RP	74%
SITE	78%
TU	38%
All (average)	65%

**Table 5.** Performance of the best predictive model per TF property, also for combinations of features and data transformation method

TF property	Feature combi-nation	Data transfor-mation	G-mean score
ACT	Lemma NER	RandomUS	0.80
DOM	Lemma NER	SVD	0.90
EVO	Lemma POS NER	SVD	0.97
RP	Lemma POS NER	SVD	0.87
SITE	NER for lemma	SVD	0.95
TU	Lemma NER	SVD	0.87

We selected as the best models those with the highest median G-mean score and with lower dispersion and skewness of performance obtained with all transformation methods (under-sampling, dimensionality reduction, and feature selection), with the goal of obtaining the most consistent predictive models. Box plots with performance in cross-validation of all trained predictive models for each TF property are shown in Supplementary Figures S2−S7. Also, detailed characteristics of the best predictive models are shown in Supplementary Table S4.

## Discussion

Our approach benefited from the large number, 177, of manually curated summaries for TFs in *E. coli*. This set the bases to implement a machine learning method whose performance, essentially recall, we could evaluate. The positive 77% result on this body of the literature provides confidence to the application of the approach we implemented, in novel collections of papers and generate, as we did, summaries for new TFs, particularly in other bacteria. As mentioned, in principle we can now apply and generate automatic summaries for TFs from different bacterial organisms with large enough available relevant publications.

Furthermore, we found that our approach is useful in two ways to assist curation of manual summaries describing TF properties. First, we extracted knowledge of properties that were absent in already-created manual summaries, since 71% of the TFs of *E. coli* gained at least one new property and our approach extracted knowledge from a median of 12 additional articles that were not utilized to write the manual summary. This new knowledge could be employed by curators of RegulonDB to improve current TF summaries. In the future, we can extend our approach to combine knowledge extraction from the literature with already-curated knowledge in databases, such as UniProt, so that we can extract ‘new knowledge’ in a broader sense. Second, we can extract knowledge to elaborate manual summaries of new bacteria, considering that in this study we generated summaries on *Salmonella* that included up to 96% of relevant knowledge according to the manual evaluation.

We expected a greater difference between the extracted knowledge of TF properties of *E. coli* when we employed the complete article collection (ROUGE-1 recall median score of 0.77) compared with the use of the TF-related article collection (ROUGE-1 recall median score of 0.67). The fact that we trained our predictive models with manual summaries can explain this small difference, since the models were ‘strict’ when selecting sentences. On the other hand, it seems appropriate to develop a strategy to create TF-related article collections for *Salmonella* before applying our automatic summarization approach. This could be achieved in the future by clustering techniques.

We succeeded in applying predictive models trained with one bacterium (*E. coli*) to extract knowledge for a different one (*Salmonella*). According to the median ROUGE-1 recall of 77% for the automatic summaries of *E. coli*, we expected nearly the same percentage of relevant sentences in the automatic summaries of *Salmonella*. This was confirmed by the manual evaluation of 10 summaries, which had up to 96% relevant sentences. Based on these results, we corroborated that our approach for knowledge extraction of TF properties can assist the curation of new summaries for different bacteria. The only prerequisites are the construction of dictionaries of the entities employed in sentence representation and the triage of articles for the target bacterium.

A limitation of our approach is that we are not fully certain that the sentences of an automatic summary refer to the TF. More elaborate approaches must be considered to more fully ensure this direct relevance, since currently we only filter those sentences that mention the TF.

Regarding the strategies employed to tackle the imbalanced data problem, we consider the G-mean the most useful aspect. This score takes into consideration the performance of the two classes, as low performance of the positive class gives a low G-mean score even if the negative class is correctly classified. Thus, the high G-mean scores obtained in supervised learning (from 0.80 to 0.97) guaranteed a correct classification of the positive class. The under-sampling techniques tested here had no positive effect when classifying, except for the classification of the ACT property.

Despite the high G-mean score of classification of the TU property (0.87), knowledge extraction of this property led to very poor results for *E. coli* and *Salmonella*. The manual evaluation by curators revealed that the predictive model learned to extract sentences with knowledge of regulation by the TF of different TUs (operons), instead of the knowledge of the TU (operon) that contains the gene encoding the TF.

We consider that the best use of the final HTML summaries generated with our approach is to use them as pieces of suggested knowledge for manual curation work, instead of directly publishable texts. Ideally, a curator should review the summaries to confirm that they can be published in a database.

## Conclusion

Here, we presented an ATS strategy based on supervised learning, for the effective knowledge extraction of TF properties from article collections. We employed this approach to generate automatic summaries of 177 TFs of *E. coli* K-12 by processing 5961 scientific articles and automatic summaries of 185 TFs of *S. typhimurium* from 3498 articles. We demonstrated that our approach can assist curation to expand already-created summaries, such as those already published in RegulonDB, and that it can assist creation of new summaries of another bacterium, such as *Salmonella*, by using a predictive model trained with manual summaries made for another, in this case, *E. coli*. We anticipate that our work can be a starting point to more sophisticated approaches to handle the accelerating body of the biomedical literature and can contribute to generate large collections of useful TF summaries from many different bacteria, contributing in this way to accelerate access to knowledge in life science research.

## Supplementary Material

baaa109_Supp
