# Supplementary material for: Knowledge extraction for assisted curation of summaries of bacterial transcription factor properties
Source: Database (Oxford). 2020 Dec 11;2020:baaa109. doi: 10.1093/database/baaa109 (PMC7731926; doi:10.1093/database/baaa109)
Supplement: baaa109_Supp [file baaa109_supp.zip › Supplementary-material_v2.docx]

Knowledge extraction for assisted curation of summaries of bacterial transcription factor properties

Supplementary material

Table S1. Specific relevant information associated to each TF property tagged in sentences of manual summaries

| Property | Specific information manually tagged | Tag |
| --- | --- | --- |
| ACT | Growth condition in negative regulation | ACTCONDN |
|  | Growth condition in positive regulation | ACTCONDP |
|  | Effector | ACTEFFE |
|  | Regulation of the TF activity | ACTREG |
|  | Active conformation of the TF | ACTCONF |
| EVO | Domain position and percentage of domain identity | EVPIDT |
|  | Percentage of TF identity with other TFs | EVPIT |
| SIT | Symmetry | SSM |
|  | Size | SSZ |
| TU | Regulation of the TU | TUR |
|  | Organization of the TU | TURO |
|  | Localization of the TU | TUL |


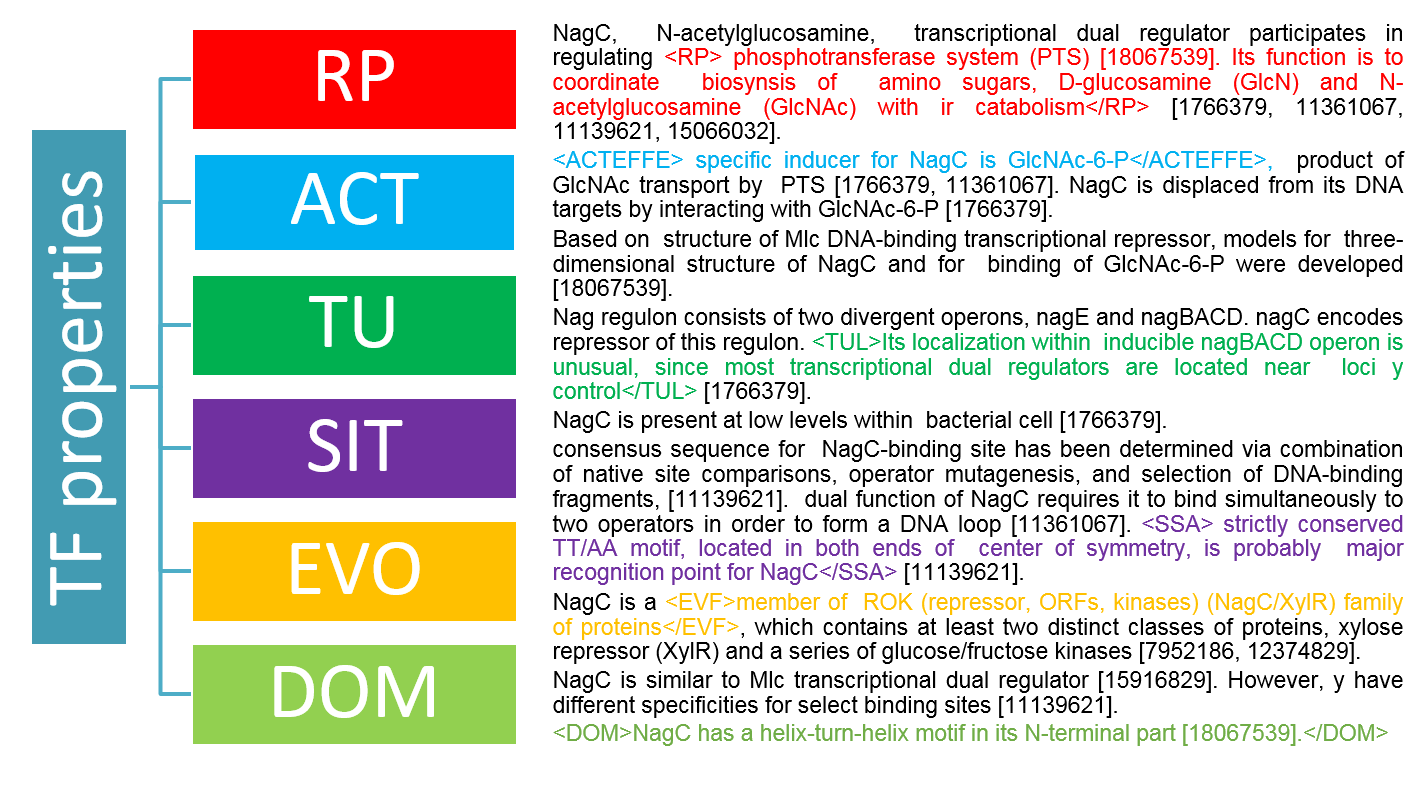


Figure S1. Example of tagged manual summary with specific relevant information.

Table S2. Biological information automatically tagged in manual summaries to enrich features for supervised learning. This information was also used to tag sentences from articles of *E. coli* and *Salmonella*. For *E. coli*, we indicate the source, and for *Salmonella* we indicate if the source was the same, the new source, or if the tag was not used

| Property | Biological information | Source for *E. coli* | Source for *Salmonella* | Tag |
| --- | --- | --- | --- | --- |
| ACT | Dictionary of TFs | RegulonDB | Team of RegulonDB^1^ | ACTTF |
|  | Dictionary of growth conditions | RegulonDB | The same | ACTCOND |
|  | Dictionary of effectors | RegulonDB | The same | ACTEFFE |
|  | Keywords of effectors | Manually collected | The same | ACTEFFE |
|  | Dictionary of regulatory verbs | Manually collected | The same | ACTREG |
|  | Keywords of regulation | Manually collected | The same | ACTREG |
|  | Dictionary of conformations | RegulonDB | Not used | ACTCONF |
|  | Keywords of conformations | Manually collected | The same | ACTCONF |
| DOM | Dictionary of structural domain families | DBD: Transcription Factor Prediction Database | The same | DFAM |
|  | Dictionary of molecular functions | GO’s OBO file | The same | MF |
|  | Dictionary of structural motifs | Interpro | The same | DMOT |
|  | Dictionary of TFs | RegulonDB | Team of RegulonDB^1^ | TF |
|  | Keywords of structural domains | Frequent words | The same | FWDOM |
| EVO | Dictionary of evolutionary families | RegulonDB | Not used | EVF |
|  | Keywords of percentage of TF identity with other TFs and percentage of domain identity | Manually collected | The same | EVPI |
|  | Dictionary of structural domain position | Manually collected | The same | EVDOM |
| RP | Dictionary of biological processes | GO | The same | PRO |
|  | Keywords of regulated processes | Frequent words | The same | FWRP |
| SIT | Keywords of symmetry | Manually collected | The same | SSM |
|  | Keywords of size | Manually collected | The same | SSZ |
|  | Spatial arrangement | Regular expression | The same | SSA |
| TU | Dictionary of genes | RegulonDB | RegulonDB and GeneBank^2^ | TURO |
|  | Dictionary of transcription units | RegulonDB | Team of RegulonDB^3^ | TURO |
|  | Keywords of organization of the TU | Manually collected | The same | TURO |
|  | Keywords of regulation of the TU | Manually collected | The same | TUR |
|  | Keywords of localization of the TU | Manually collected | The same | TUL |

^1^ Predicted by E. Pérez-Rueda based on Perez-Rueda, E., Tenorio-Salgado, S., Huerta-Saquero, A., Balderas-Martínez, Y. I. & Moreno-Hagelsieb, G. The functional landscape bound to the transcription factors of Escherichia coli K-12. Comput Biol Chem 58, 93–103 (2015).

^2^ <https://www.ncbi.nlm.nih.gov/nuccore/NC_003197.2>.

^3^ Predictions based on Salgado, H., Moreno-Hagelsieb, G., Smith, T. F. & Collado-Vides, J. Operons in Escherichia coli: genomic analyses and predictions. Proc Natl Acad Sci USA 97, 6652–6657 (2000); Moreno-Hagelsieb, G. & Collado-Vides, J. A powerful non-homology method for the prediction of operons in prokaryotes. Bioinformatics 18 Suppl 1, S329–36 (2002); and Moreno-Hagelsieb, G. Operons Across Prokaryotes: Genomic Analyses and Predictions 300 Genomes Later. Current Genomics 7, 163–170 (2006).

Table S3. A general description of the experimental setup, including the tested values of the different aspects employed for training the six classifiers

| Aspect | Values |
| --- | --- |
| Combination of features | lemma POS NER, lemma NER, NER for lemma |
| Vectorizer | Binary, TF-IDF, TF-IDF binary |
| N-grams | 1, 1-2, 1-3 |
| Dimensionality reduction with SVD | 300, 200 dimensions |
| Feature selection with $x^{2}$ | 1000, 800, 500 features |
| Under-sampling technique | RandomUS, Tomek, IHT, and OSS |
| SVM kernel | rbf, lineal, poly |
| Class weighting | true, false |


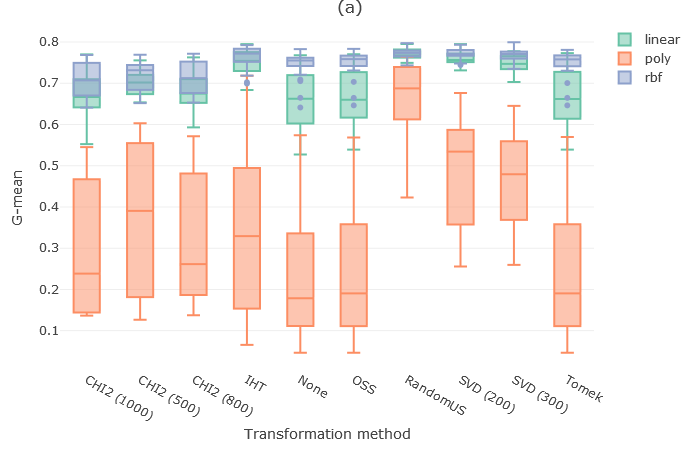

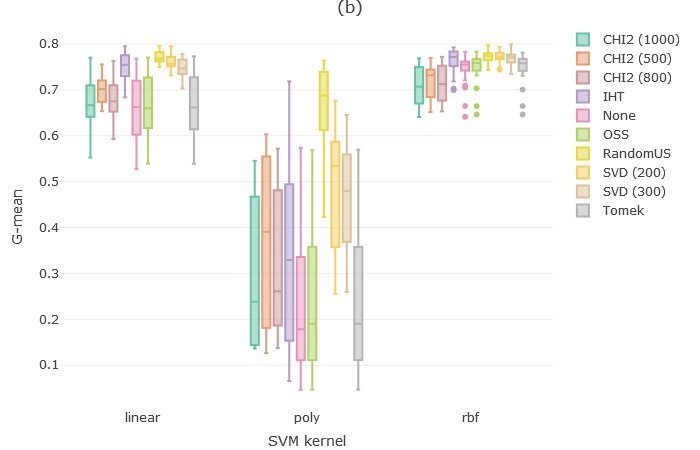


Figure S2. Distribution of performance in cross-validation of all trained predictive models for ACT property. (a) shows transformation methods in horizontal axis, CHI2 ($\chi^{2}$) and SVD include number of final dimensions/features. (b) shows SVM kernel in horizontal axis.


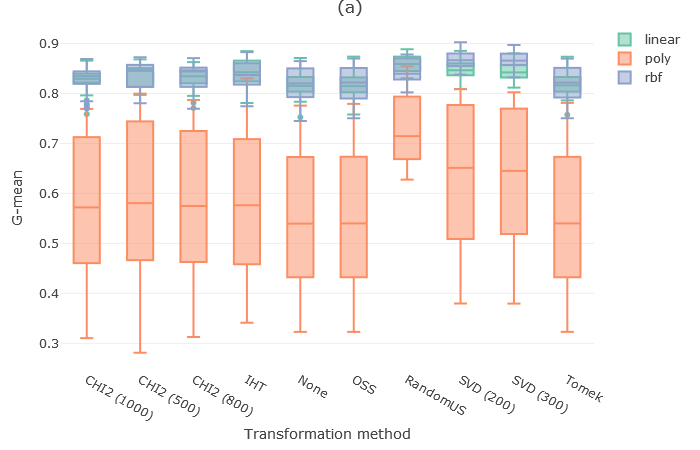

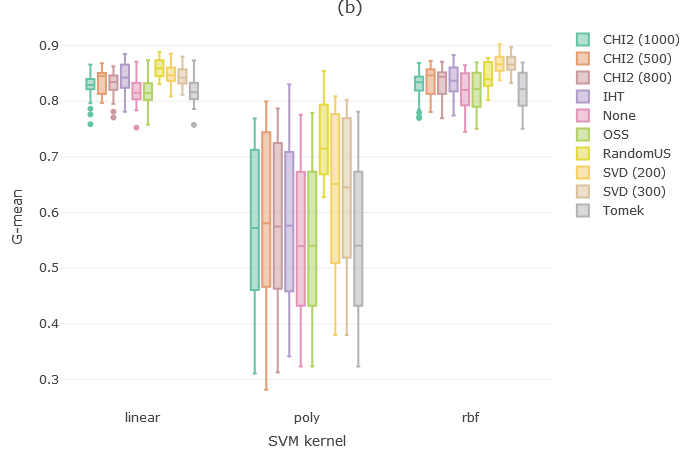


Figure S3. Distribution of performance in cross-validation of all trained predictive models for DOM property. (a) shows transformation methods in horizontal axis, CHI2 ($\chi^{2}$) and SVD include number of final dimensions/features. (b) shows SVM kernel in horizontal axis.


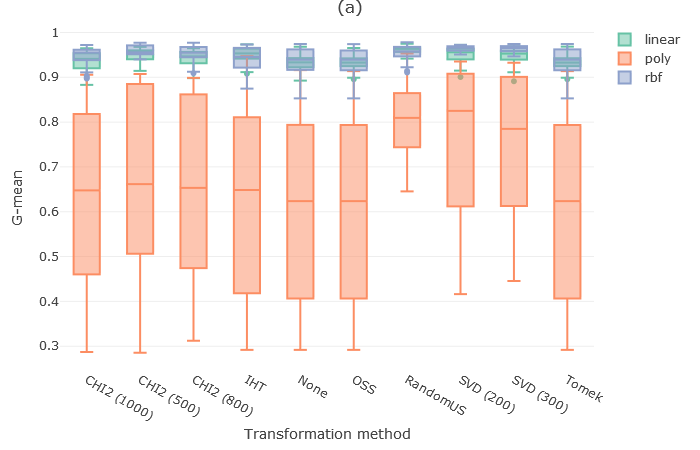

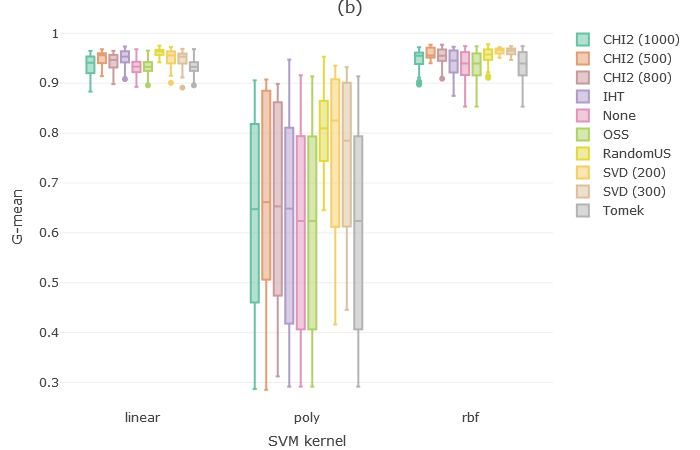


Figure S4. Distribution of performance in cross-validation of all trained predictive models for EVO property. (a) shows transformation methods in horizontal axis, CHI2 ($\chi^{2}$) and SVD include number of final dimensions/features. (b) shows SVM kernel in horizontal axis.


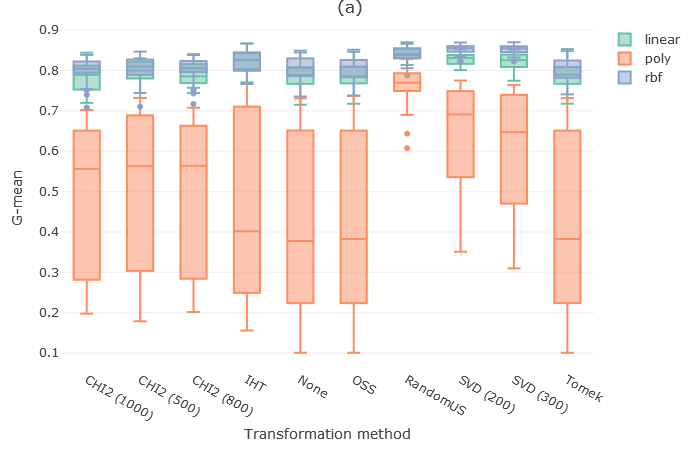

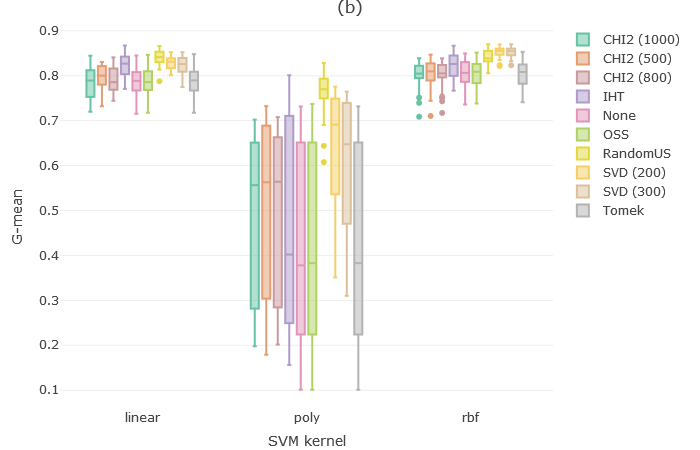


Figure S5. Distribution of performance in cross-validation of all trained predictive models for RP property. (a) shows transformation methods in horizontal axis, CHI2 ($\chi^{2}$) and SVD include number of final dimensions/features. (b) shows SVM kernel in horizontal axis.


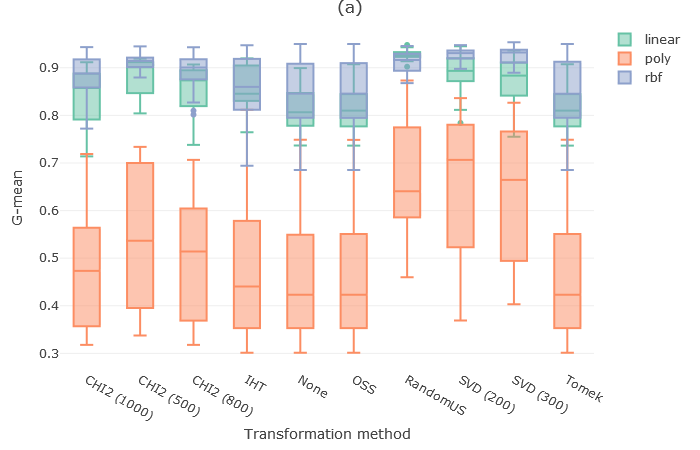

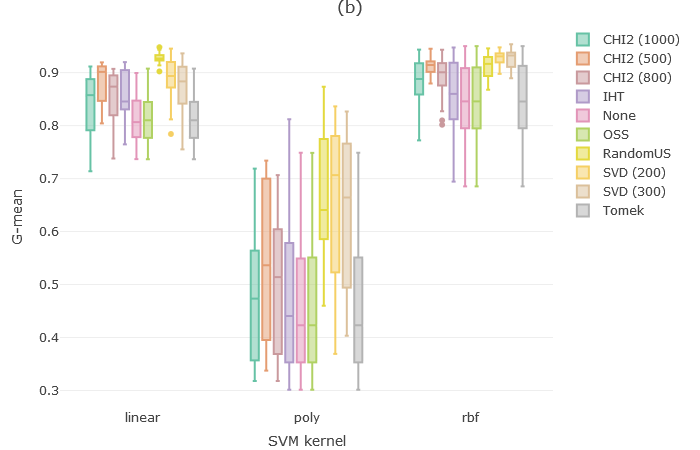


Figure S6. Distribution of performance in cross-validation of all trained predictive models for SIT property. (a) shows transformation methods in horizontal axis, CHI2 ($\chi^{2}$) and SVD include number of final dimensions/features. (b) shows SVM kernel in horizontal axis.


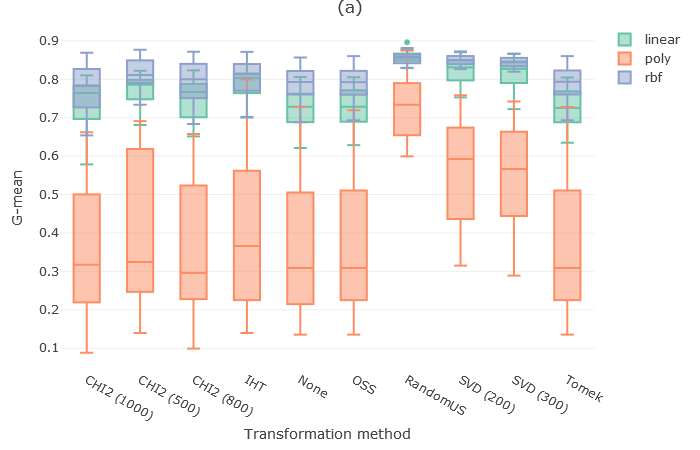

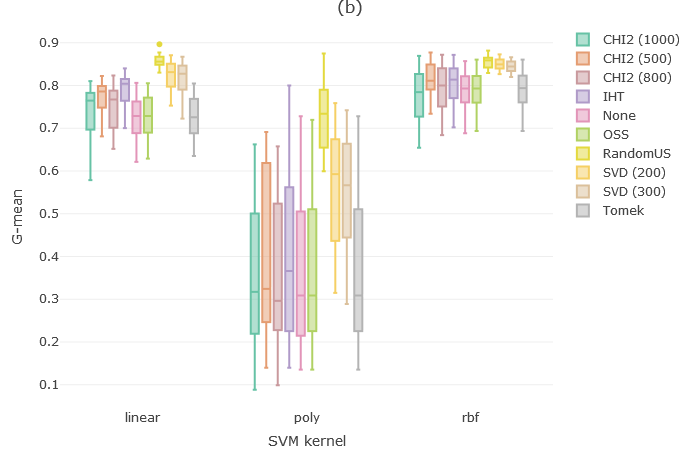


Figure S7. Distribution of performance in cross-validation of all trained predictive models for TU property. (a) shows transformation methods in horizontal axis, CHI2 ($\chi^{2}$) and SVD include number of final dimensions/features. (b) shows SVM kernel in horizontal axis.

Table S4. Detailed characteristics of the best predictive model per TF property

| Property | Vectorization | N-  grams | Transformation | Final dimensions | SVM hyperparameters | | | | G-mean score |
| --- | --- | --- | --- | --- | --- | --- | --- | --- | --- |
|  |  |  |  |  | Kernel | C | Gamma | Class weight |  |
| ACT | TF-IDF | 1,2 | RandomUS | -- | RBF | 3.0 | 1.0 | Balanced | 0.80 |
| DOM | TF-IDF | 1 | SVD | 200 | RBF | 1.0 | 1.0 | Balanced | 0.90 |
| EVO | TF-IDF binary | 1 | SVD | 200 | RBF | 0.5 | 1.0 | Balanced | 0.97 |
| RP | TF-IDF binary | 1,2 | SVD | 200 | RBF | 1.0 | 1.0 | Balanced | 0.87 |
| SIT | TF-IDF binary | 1 | SVD | 200 | RBF | 0.5 | 1.0 | Balanced | 0.95 |
| TU | TF-IDF binary | 1 | SVD | 200 | RBF | 3.0 | 0.1 | Balanced | 0.87 |
